# Methods and Models for Diagnosis and Prognosis in Medical Systems

**DOI:** 10.1155/2013/184257

**Published:** 2013-10-03

**Authors:** Angel García-Crespo, Giner Alor-Hernandez, Linamara Battistella, Alejandro Rodríguez-González

**Affiliations:** ^1^Computer Science Department, Universidad Carlos III de Madrid, Avenida de la Universidad 30, 28911 Leganés, Spain; ^2^Division of Research and Postgraduate Studies, Instituto Tecnológico de Orizaba, Avenida Oriente 9, 852 Colonia Emiliano Zapata, CP 94320 Orizaba, VER, Mexico; ^3^Medical School, Universidade de São Paulo, Avenida Dr. Arnaldo, 455 Cerqueira César, 01246903 São Paulo, SP, Brazil; ^4^Bioinformatics at Centre for Plant Biotechnology and Genomics UPM-INIA, Pozuelo de Alarcón, Parque Científico y Tecnológico, UPM, Campus de Montegancedo, Carretera M-40, km 38, 28223 Madrid, Spain

## 1. Introduction

During years, the development of medical systems (as part of Clinical Decision Support Systems (CDSS)) has been one of the main research fields in biomedical informatics area. Different systems have been proposed to improve the quality of medical practice in several different areas such as pharmacy management, electronic health records, diagnosis systems, telemedicine, and medical imaging among others.

Several approaches and models have been developed since, the earliest 60s. Also several methods and techniques from the fields of artificial intelligence, decision theory, and statistics have been introduced into models of the medical management of patients (diagnosis, treatment, and follow-up); in some of these models, assessment of the expected prognosis constitutes an integral part. Typically, recent prognostic methods rely on explicit (patho)physiological models, which may be combined with traditional models of life expectancy.

The main goals of this special issue were the publication of new algorithms and mathematical methods, decision theories, techniques, and models based on probabilistic and quantitative approaches to solve existing or new issues.

## 2. Papers

The impact of the special issue comes from the submission of twenty-four research papers. From these twenty-four papers, only twelve were finally accepted for publication in this special collection. The contributions were selected based on their innovation and quality, demonstrating their applicability and importance in the field. A brief description of each selected paper for this collection is presented in the following.

In paper *“Normality index of ventricular contraction based on a statistical model from FADS”* by L. Jiménez-Ángeles et al., the authors present a probability density function model of the 3 most significant factors present in a dynamic series of equilibrium radionuclide angiography images extracted from factor analysis of dynamic structures for a control group which is presented. The authors also present an index, based on the likelihood between the control group's contraction model and a sample of normal subjects. The proposed index provides a measure, consistent with the phase analysis currently used in clinical environments.

Paper *“Development of an expert system as a diagnostic support of cervical cancer in atypical glandular cells, based on fuzzy logics and image interpretation”* by K. R. Domínguez-Hernández, et al. describes the development of an expert system which can provides a diagnosis to cervical neoplasia precursors injuries through the integration of fuzzy logics and image interpretation techniques.

The paper, *“The iOSC3 system: using ontologies and SWRL rules for intelligent supervision and care of patients with acute cardiac disorders”* by M. Martínez-Romero et al., presents the design, development, and validation of iOSC3, an ontology-based system for intelligent supervision and treatment of critical patients with acute cardiac disorders.

In paper “*An intelligent system approach for asthma prediction in symptomatic preschool children”* by Chatzimichail et al., the authors present a study where a new method for asthma outcome prediction based on principal component analysis and least square support vector machine classifier is proposed.

In paper *“Ontology-oriented diagnostic system for traditional chinese medicine based on relation refinement”* by P. Gu et al., the authors define the diagnosis in traditional Chinese medicine as the discovery of fuzzy relations between symptoms and syndromes. The authors created an ontology-oriented diagnostic system to address the knowledge-based diagnosis through relation refinement based on a well-defined ontology of syndromes.

In paper *“Evaluation of the diagnostic power of thermography in breast cancer using Bayesian network classifiers,”* C.-R. Nicandro et al. evaluate the diagnostic power of thermography in breast cancer using Bayesian network classifiers. The authors show how the information provided by the thermal image can be used to characterize patients suspected of having cancer.

The paper *“Detection of structural changes in tachogram series for the diagnosis of atrial fibrillation events,”* by F. Ieva et al. presents a new statistical method to deal with the identification of atrial fibrillation events based on the order of identification of the ARIMA models used for describing the RR time series that characterize the different phases of atrial fibrillation (before-, during, and after-AF).

The paper *“Study of the effect of breast tissue density on detection of masses in mammograms,”* A. García-Manso et al. study the effect of BI-RADS density in their project for developing an image-based CAD system to detect masses in mammograms.

The paper *“Mobile personal health system for ambulatory blood pressure monitoring”* by L. J. Mena et al. presents ARV mobile, a multiplatform mobile personal health monitor (PHM) application for ambulatory blood pressure (ARP) monitoring that has the potential to aid in the acquisition and analysis of detailed profile of ABP and heart rate (HR), improving the early detection and intervention of hypertension, offering a tailored hypertension control, allowing also and detecting potential abnormal BP and HR levels for timely medical feedback.

The paper *“Statistical evaluation of a fully automated mammographic breast density algorithm”* M. by Abdolell et al. presents a study of the statistical evaluation of a fully automated, area-based mammographic density measurement algorithm. This algorithm is behind the idea that visual assessments of mammographic breast density by radiologist are used in clinical practice; however, these assessments have shown weaker associations with breast cancer risk than area-based quantitative methods.

The paper *“Locomotor development prediction based on statistical model parameters identification”* by A. Wildemann et al. presents and introduces an approach for parameter identification on a statistical prediction model by means of the use of available individual data. The application of the method was done for predicting the movement of a patient with congenital motility disorders.

Finally, the paper *“Design and customization of telemedicine systems”* by C. I. Martínez-Alcalá et al. is focused on the improvement of telemedicine systems with the aim of customizing therapies according to the profile and disability of patients. The work proposes the adoption of user-centered design methodology for the design and development of telemedicine systems in order to support the rehabilitation of patients with neurological disorders.

## 3. Conclusions

As can be seen, all the papers accepted have a direct relation with the scope of the special issue, and all of them provide quite interesting research techniques, models, and studies directly applied to the area of medical diagnostic systems.

*Angel García-Crespo* *Giner Alor-Hernandez* *Linamara Battistella* *Alejandro Rodríguez-González*

